# Supplementary material for: A Bayesian analysis of the association between Leukotriene A4 Hydrolase genotype and survival in tuberculous meningitis
Source: eLife. 2021 Jan 8;10:e61722. doi: 10.7554/eLife.61722 (PMC7793626; doi:10.7554/eLife.61722)
Supplement: Supplementary file 1. [file elife-61722-supp1.doc]

**Table S1. Characteristics of included and excluded Indonesia patients**

|  | **included** | **excluded** |
| --- | --- | --- |
| **Total** | 376 | 139 |
| **Age in years**  ***median (range)*** | 28 (14-90) | 30 (15-68) |
| **Glasgow Coma Score**  ***median (range)*** | 13 (5-15) | 14 (3-15)  *unknown* = 62/139 (44.6%) |
| **BMRC TBM grade**  ***no. (% of total)***  **1**  **2**  **3** | 34 (9.0)  284 (75.5)  58 (15.4) | 17 (22.1)  45 (58.4)  15 (19.5)  *unknown* = 62/139 (44.6) |
| **Overall mortality** ***no. (%)*** | 146 (39.9) | 58 (46.4)  *unknown* = 14/139 (10.1) |
| **rs17525495 *LTA4H***  ***no. (% total)***  **CC**  **CT**  **TT** | 216 (57.5)  128 (34.0)  32 (8.5) | 24 (46.2)  24 (46.2)  4 (7.7)  *unknown* = 87/139 (62.6) |
