# Supplementary material for: A Bayesian analysis of the association between Leukotriene A4 Hydrolase genotype and survival in tuberculous meningitis
Source: eLife. 2021 Jan 8;10:e61722. doi: 10.7554/eLife.61722 (PMC7793626; doi:10.7554/eLife.61722)
Supplement: Transparent reporting form [file elife-61722-transrepform.pdf]

Westbrook Centre, Milton Road
Cambridge CB4 1YG
UK
P 01223 855340
W elifesciences.org
T @elife

eLife's transparent reporting form

We encourage authors to provide detailed information *within their submission* to facilitate the interpretation and replication of experiments. Authors can upload supporting documentation to indicate the use of appropriate reporting guidelines for health-related research (see EQUATOR Network), life science research (see the BioSharing Information Resource), or the ARRIVE guidelines for reporting work involving animal research. Where applicable, authors should refer to any relevant reporting standards documents in this form.

If you have any questions, please consult our Journal Policies and/or contact us: editorial@elifesciences.org.

Sample-size estimation

- You should state whether an appropriate sample size was computed when the study was being designed
- You should state the statistical method of sample size computation and any required assumptions
- If no explicit power analysis was used, you should describe how you decided what sample (replicate) size (number) to use

Please outline where this information can be found within the submission (e.g., sections or figure legends), or explain why this information doesn't apply to your submission:

This paper is a re-analysis of survival data using Bayesian inference methods from two published cohorts, Thuong et al. and van Laarhoven et al., both 2017 JID. This is made explicit throughout the paper. All individuals for whom survival data and LTA4H genotyping data were available were used. Since we had no control over the number of available patients, a power analysis is not appropriate.

Replicates

- You should report how often each experiment was performed
- You should include a definition of biological versus technical replication
- The data obtained should be provided and sufficient information should be provided to indicate the number of independent biological and/or technical replicates
- If you encountered any outliers, you should describe how these were handled
- Criteria for exclusion/inclusion of data should be clearly stated
- High-throughput sequence data should be uploaded before submission, with a private link for reviewers provided (these are available from both GEO and ArrayExpress)

Please outline where this information can be found within the submission (e.g., sections or figure legends), or explain why this information doesn't apply to your submission:

This information is given at the start of the methods section in so far as it is applicable. An Excel spreadsheet with all patient data is included.

eLife Sciences Publications, Ltd is a limited liability non-profit non-stock corporation incorporated in the State Delaware, USA, with company number 5030732, and is registered in the UK with company number FC030576 and

Statistical reporting

- Statistical analysis methods should be described and justified
- Raw data should be presented in figures whenever informative to do so (typically when N per group is less than 10)
- For each experiment, you should identify the statistical tests used, exact values of N, definitions of center, methods of multiple test correction, and dispersion and precision measures (e.g., mean, median, SD, SEM, confidence intervals; and, for the major substantive results, a measure of effect size (e.g., Pearson's r, Cohen's d)
- Report exact p-values wherever possible alongside the summary statistics and 95% confidence intervals. These should be reported for all key questions and not only when the p-value is less than 0.05.

Please outline where this information can be found within the submission (e.g., sections or figure legends), or explain why this information doesn't apply to your submission:

The methods are described and justified in full detail in the supplementary methods section.

Raw data are presented in an Excel spreadsheet.

This is a Bayesian analysis that generates posterior distributions of probabilities of survival and hazard rates for mortality. Figures show the mean posterior probability of survival or hazard rate respectively, as well as an equi-tailed Bayesian confidence interval (credible interval) at each time point. In addition, probabilities for which of two probabilities of survival (respectively hazard rate) is greater are given for each comparison of subsets of patients. Numbers of patients in each subset are shown in each figure. Frequentist p-values are not appropriate in a Bayesian study.

All this is made clear in Box 3 and is clearly stated in the there-defined abbreviated form in the figure legends throughout the paper.

The issue of multiple comparisons is addressed in Box 1 and in the text referring to it in the penultimate two paragraphs of the Introduction.

(For large datasets, or papers with a very large number of statistical tests, you may upload a single table file with tests, Ns, etc., with reference to sections in the manuscript.)

Group allocation

- Indicate how samples were allocated into experimental groups (in the case of clinical studies, please specify allocation to treatment method); if randomization was used, please also state if restricted randomization was applied
- Indicate if masking was used during group allocation, data collection and/or data analysis

Please outline where this information can be found within the submission (e.g., sections or figure legends), or explain why this information doesn't apply to your submission:

This paper is a reanalysis of two prior clinical studies. Answers to these questions are published in the two papers cited throughout our paper.

Additional data files ("source data")

- We encourage you to upload relevant additional data files, such as numerical data that are represented as a graph in a figure, or as a summary table
- Where provided, these should be in the most useful format, and they can be uploaded as "Source data" files linked to a main figure or table
- Include model definition files including the full list of parameters used
- Include code used for data analysis (e.g., R, MatLab)
- Avoid stating that data files are "available upon request"

Please indicate the figures or tables for which source data files have been provided:

Patient data are uploaded as an Excel spreadsheet.

Matlab code is uploaded as a compressed tarball.

Parameters used are listed in the Supplementary Methods section as well as in the parameter-setting file setpriorcoords.m in uploaded in the compressed tarball of Matlab code.
